# Long non-coding RNA SNHG12 promotes immune escape of ovarian cancer cells through their crosstalk with M2 macrophages

**DOI:** 10.18632/aging.103653

**Published:** 2020-09-14

**Authors:** Min Qian, Wanwen Ling, Zhengyi Ruan

**Affiliations:** 1 Department of Obstetrics and Gynecology, Shanghai Ninth People’s Hospital, School of Medicine, Shanghai Jiaotong University, Shanghai 200011, P. R. China; 2 Equal contribution

**Keywords:** ovarian cancer, M2 macrophage, immune escape, long noncoding RNA-small nucleolar RNA host gene 12, interlukin-6

## Abstract

Accumulating evidence shows that the tumor microenvironment contributes to this phenomenon and that long non-coding RNAs (lncRNAs) are also involved in this process. In this study, we identified a new lncRNA small nucleolar RNA host gene 12 (SNHG12) and investigated its role in tumor immune escape. We analyzed the expression levels of interlukin (IL)-6R and programmed death-ligand 1 (PD-L1) in 51 ovarian cancer and 20 normal specimens by immunohistochemistry. The correlation between SNHG12 and IL-6R in clinical ovarian cancer samples was identified by RT-qPCR. We then performed SNHG12 gain- and loss-function experiments in order to investigate its role in the regulation of immune escape and the crosstalk between miR-21 and IL-6. T cell proliferation was assessed by flow cytometry. *In vivo* pro-immune escape activity of SNHG12 was assessed by tumor-xenograft mouse model. IL-6R and PD-L1 were found to be overexpressed in clinical ovarian cancer specimens. Meanwhile, SNHG12 and IL-6R expressions were positively correlated in clinical ovarian cancer samples. SNHG12 facilitated ovarian immune escape by promoting IL-6/miR-21 crosstalk between ovarian cancer cells and M2 macrophages. Notably, SNHG12 promoted IL-6R transcription by recruiting NF-κB1 to the IL-6R promoter. Our study reveals that SNHG12 facilitates ovarian cancer immune escape by upregulating IL-6R.

## INTRODUCTION

Ovarian cancer is the fifth leading cause of cancer-related deaths in women worldwide; patients are often diagnosed in the late stages leading to poor diagnosis and high relapse rate [1]. Data show that about 7%-10% of ovarian cancers are due potentially to an inherited genetic component [2]. A large number of ovarian cancer patients relapse and die within several years after the initial remission, in large part due to developing resistance to chemotherapy [3]. Thus, additional therapeutic methods for ovarian cancer are urgently needed. Previous data show that ovarian cancer expresses immunogenic tumor-associated antigens, pointing to immunotherapy as a promising therapeutic approach [4]. However, unlike in several other cancers such as non-small cell lung cancer, immunotherapy has only modest effects in ovarian cancer cases [5]. Further investigation revealed that ovarian cancers maintain a highly immunosuppressive tumor microenvironment which might explain why immunotherapy efficiency is limited [6].

Extracellular vesicles (EVs) represent a class of membrane-enclosed, nano-sized, cellular vesicles and are secreted from various cell types, including normal and pathological cells [7]. EVs are recognized as an important mediator of cell-cell communication through the transfer of proteins, lipids, and nucleic acids (mRNA, ncRNAs and DNA) [8]. A recent study revealed that EVs isolated from ovarian cancer patients were able to suppress NFAT, CD69 and CD107a expression and TCR-dependent nuclear translocation of NF-κB, with an end result of inhibiting T-cell proliferation and cytokine production [9]. In addition, small EVs secreted from ovarian cancer cells can inhibit T-cell responses and promote cancer growth [10]. Increasing evidence demonstrated that the crosstalk between tumor and immune system cells plays a vital role in tumor immune escape. IL-6 secreted by immune cells stimulated PD-L1 and miR-21 expression in colorectal cancer cells. In addition, miR-21 could be packed into EVs and secreted by tumor cells in order to generate more IL-6 secretion from immune cells. This crosstalk promotes tumor immune escape and growth [11]. LncRNAs are long RNA transcripts without protein coding capacity [12]. In this study, we performed gain- and loss- of function experiments to reveal the role of the small nucleolar RNA host gene 12 (SNHG12) in immune escape by generating the crosstalk between ovarian cancer cells and M2 macrophages.

## RESULTS

### IL-6 secreted by M2 macrophages induced the expression of PD-L1 and miR-21 in ovarian cancer cells

**Figure 1:**
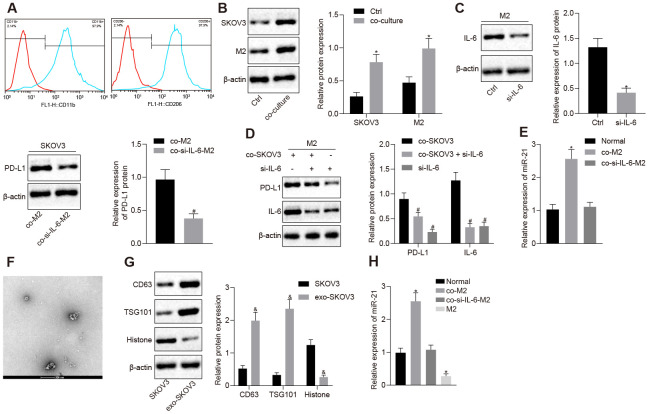
**IL-6 from M2 macrophage elevates the expression of PD-L1 and miR-21 in ovarian cancer cells.** (**A**) The markers of M2 macrophage, CD206 and CD11b, were measured by flow cytometry. (**B**) Protein level of PD-L1 after SKOV3 and M2 macrophage co-culture, in both cell types detected by western blotting. (**C**) The change of PD-L1 expression after SKOV3 co-culture with IL-6 depleted M2 macrophages was detected by western blotting. (**D**) The expression of PD-L1, IL-6 and PD-L1 were determined by Western blotting analysis. (**E**) The expression of miR-21 after SKOV3 co-cultured with IL-6 depleted M2 macrophages was determined by RT-qPCR. (**F**) Secretion of EVs was observed by TEM. (**G**) The markers for EVs isolated from SKOV3 were determined by Western blotting analysis. (**H**) The level of miR-21 in EVs isolated from SKOV3 co-cultured with IL-6 depleted M2 macrophages was assessed by RT- qPCR. * *p* < 0.05 *vs.* control or normal. # *p* < 0.05 *vs.* co-M2 or co-SKOV3. & *p* < 0.05 *vs.* SKOV3. Statistical comparisons were performed using unpaired t test when only two groups were compared or by Tukey’s test-corrected one-way ANOVA with when more than two groups were compared.

Previous studies [13–15] showed that high levels of IL-6 were found in the blood and ascitic fluid of ovarian cancer patients. To investigate the effect of IL-6 secreted by M2 macrophage on immune escape, we induced the polarization of M2 macrophages and confirmed it via classic M2 macrophage markers, CD206 and CD11b, detected through flow cytometry (Figure 1A). Next, we looked at PD-L1 expression by Western blotting analysis, and found PD-L1 was increased in both M2 macrophages and the ovarian cancer cell line SKOV3, after M2 macrophages and SKOV3 had been co-cultured (Figure 1B, 1C, *p* < 0.05). Next, to verify whether the high expression of PD-L1 was mediated by IL-6, SKOV3 was co-cultured with M2 macrophage in which IL-6 had been depleted. Western blotting analysis showed that inhibition of IL-6 could reverse the overexpression of PD-L1 in both cell types, indicating that IL-6 secretion was the key for PD-L1 upregulation (Figure 1D, *p* < 0.05). A positive feedback between IL-6 and miR-21 has been reported, where IL-6 secreted by M2 macrophages induces miR-21 expression in cancer cells, leading to the release of EVs packed with miR-21 which acts on the M2 macrophages further increasing the secretion of IL-6 [11]. Meanwhile, both miR-21 and IL-6 had been reported to be involved in regulation of PD-L1. Thus, we hypothesized miR-21/IL-6 crosstalk might be involved in PD-L1 regulation. We found that the expression of miR-21 in SKOV3 was dramatically increased after co-culture with M2 macrophages and, upregulated miR-21 could be restored after IL-6 depletion (Figure 1E, *p* < 0.05). This result suggested that IL-6 secreted by M2 macrophage could regulate miR-21 expression in SKOV3. Meanwhile, TEM results showed SKOV3 cells could secrete extracellular vesicles (EVs) (Figure 1F), a finding that we further confirmed with the EV markers CD63 and TGS101 (Figure 1G, *p* < 0.05). Next, we analyzed the miR-21 expression in M2 macrophage condition media (CM) and found very low expression of miR-21. Interestingly, miR-21 expression was significantly increased in SKOV3 CM after culture in M2 macrophage CM. Furthermore, silencing IL-6 in M2 macrophages abolished the upregulation of miR-21 in SKOV3 CM after culture in M2 macrophage CM (Figure 1H, *p* < 0.05). Collectively, our data revealed that IL-6 secreted by M2 macrophages mediated PD-L1 and miR-21 expression in the ovarian cancer cell line SKOV3.

### miR-21-containing EVs secreted by ovarian cancer cells increased secretion of IL6 from M2 macrophages leading to high levels of PD-L1 in both cells

**Figure 2:**
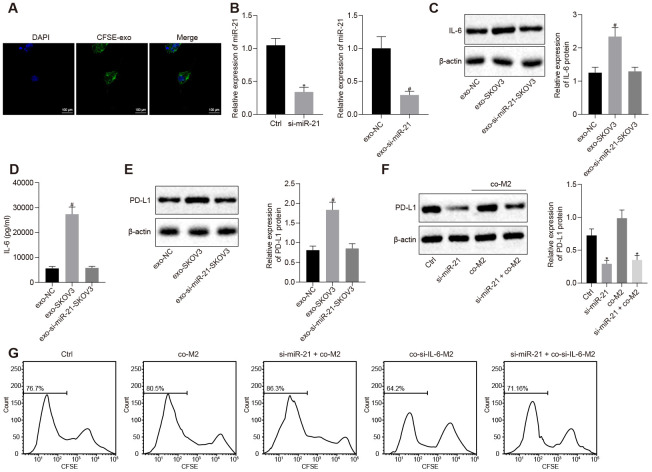
**miR-21 secreted from ovarian cancer cells facilitates the secretion of IL-6 from M2 macrophages and this crosstalk elevates the expression of PD-L1 in both cell lines.** (**A**) EVs uptake capacity of M2 macrophage visualized by CFSE-labeled EVs. (**B**) knock down efficiency of miR-21 assessed by RT-qPCR. (**C**) effect of miR-21 in EVs on the expression of IL-6 in M2 macrophage determined by Western blotting and RT-qPCR. (**D**) secretion of IL-6 by M2 macrophages was determined by ELISA assay. (**E**, **F**) expression of PD-L1 in M2 macrophage and SKOV3 determined by Western blotting analysis and RT-qPCR. (**G**) proliferation of CFSE-labeled T cells was assessed by flow cytometry. * *p* < 0.05 *vs.* control or co-M2. # *p* *<* 0,05 *vs.* EVs-NC. Statistical comparisons were performed using unpaired t test when only two groups were compared or by Tukey’s test-corrected one-way ANOVA with when more than two groups were compared.

To investigate the effect of miR-21 containing EVs from ovarian cancer cells on IL-6 expression from M2 macrophages, we extracted and CFSE labeled EVs from SKOV3 and found that they are able to enter M2 macrophages (Figure 2A). Furthermore, the expression and secretion of IL-6 were dramatically increased in the presence of EVs from SKOV3. However, after silencing miR-21 (Figure 2B, *p* < 0.05), the IL-6 expression in M2 macrophages remained unchanged (Figure 2C, 2D, *p* < 0.05), indicating that miR-21 was indeed responsible for the induction of IL-6 in M2 macrophages. Given that IL-6 was critical for the induction of PD-L1, we also investigated the role of miR-21 in PD-L1 regulation. We co-cultured M2 macrophages with control or miR-21 silenced SKOV3 cells, and found that PD-L1 was significantly downregulated in both cells after depletion of miR-21 (Figure 2E, 2F, *p* < 0.05). Furthermore, when miR-21 was silenced in SKOV3 or IL-6 was depleted in M2 macrophages, the growth inhibition of T cells co-cultured with SKOV3 was removed (Figure 2G, *p* < 0.05). Taken together, these data indicated that IL-6 secreted by M2 macrophage along with miR-21 present in SKOV3 derived EVs, inhibited T cell proliferation by upregulating PD-L1.

### IL-6R facilitated PD-L1 expression in response to miR-21/IL-6 crosstalk

**Figure 3:**
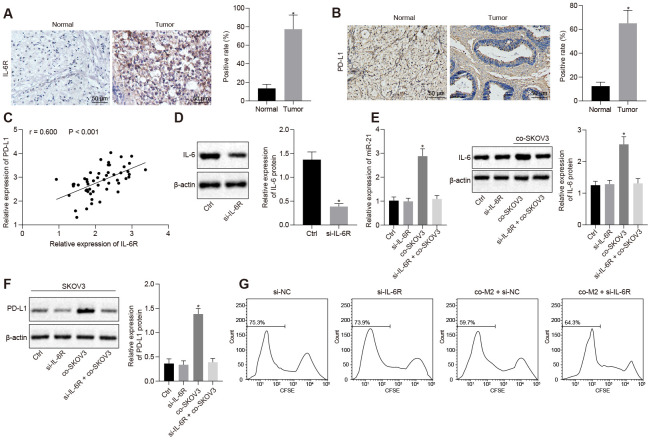
**Overexpressed IL-6R in SKOV3 response to miR-21/IL-6 crosstalk elevates expression of PD-L1.** (**A**, **B**) The expression of IL-6R and PD-L1 in ovarian cancer biopsy specimens analyzed by IHC. (**C**) correlation between IL-6R and PD-L1. (**D**) knock down efficiency of IL-6R assessed by western blotting. (**E**) expression of miR-21 after depletion of IL-6R determined by RT-qPCR. (**F**) change of PD-L1 after IL-6R silencing assessed by Western blotting analysis. (**G**) proliferation of T cells after inhibition of IL-6R analyzed by flow cytometry. * *p* < 0.05 *vs.* control or normal. Statistical comparisons were performed using unpaired t test when only two groups were compared or by Tukey’s test-corrected one-way ANOVA with when more than two groups were compared.

We assumed IL-6R, a receptor for IL-6, might play a role in the regulation of PD-L1 by miR-21/IL-6 axis. We detected IL-6R expression in clinical ovarian cancer patient’s sample by IHC and found that IL-6R expression was much higher in ovarian cancer tissues (Figure 3A, *p* < 0.05), which might indicate that high levels of IL-6R would be required for PD-L1 induced via the miR-21/IL-6 feedback loop. Similarly, the expression of PD-L1 was also higher in ovarian cancer tissues (Figure 3B, *p* < 0.05). Meanwhile, IL-6R expression was positively correlated with PD-L1 expression (Figure 3C, *p* < 0.05). Furthermore, both the induction of IL-6 in M2 macrophages and the release of miR-21 containing EVs from SKOV3 were diminished after M2 macrophages were co-cultured with SKOV3 cells where IL-6R has been knocked down (Figure 3D, 3E, *p* < 0.05). At the same time, PD-L1 expression was reduced once M2 macrophages were co-cultured with IL-6R depleted SKOV3 cells (Figure 3F). As a consequence, growth inhibition of T cells caused by co-culture with SKOV3, which had been co-cultured with M2 macrophages in advance, was rescued by depletion of IL-6R (Figure 3G). Collectively, our data revealed IL-6R as a receptor of IL-6 mediated the regulation of PD-L1 by interrupting the miR-21/IL-6 crosstalk, thus promoting ovarian cancer immune escape.

### SNHG12 was positively regulated IL-6R by NF-κB1

**Figure 4:**
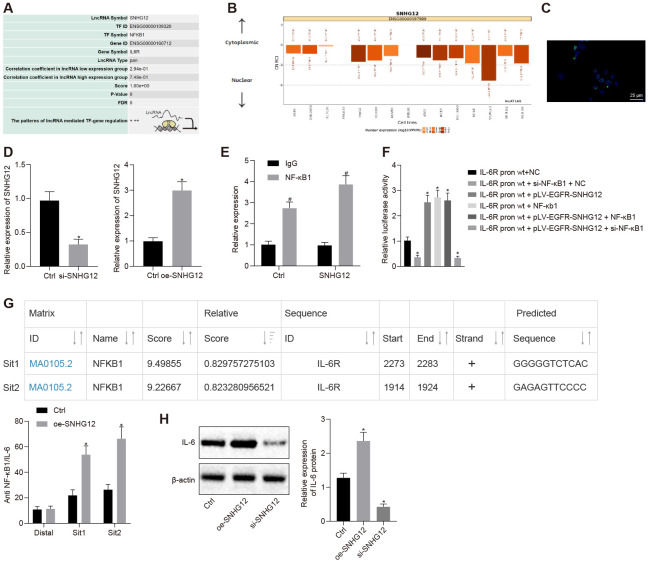
**SNHG12 facilitates the expression of IL-6R by NF-κB1.** (**A**) Transcription factor for upregulation of IL-6R by SNHG12 predicted by the LNCmap database. (**B**) sub-localization of SNHG12 predicted by the LNCmap database. (**C**) sub-localization of SNHG12 visualized by FISH (**D**) expression of SNHG12 detected by RT- qPCR. (**E**) the binding of SNHG12 and NF-κB1 determined by RIP. (**F**) effects of SNHG12 and NF-κB1 on IL-6R promoter activity assessed by dual luciferase assay. (**G**) The binding of NF-κB1 to IL-6R promoter determined by ChIP. (**H**) the expression of IL-6R after silencing or overexpressing SNHG12 determined by Western blotting analysis. * *p* < 0.05 *vs.* control or IL-6R-WT + NC. # *p* < 0.05 *vs.* IgG. Statistical comparisons were performed using unpaired t test when only two groups were compared or by Tukey’s test-corrected one-way ANOVA with when more than two groups were compared.

To better understand how IL-6R expression is upregulated in ovarian cancer cells, we investigated its transcriptional regulation. NF-κB1 is a well-known master transcription factor, which also regulates the IL-6R gene and, in addition, the lncMAP database predicted the SNHG12 to localize in nucleus (Figure 4A, 4B). Indeed, SNHG12 was localized in the nucleus according to the results of our FISH experiment (Figure 4C). Next, we knocked down and overexpressed SNHG12 (Figure 4D, *p* < 0.05). Meanwhile, a RIP assay showed the direct interaction between SNHG12 and NF-κB1 (Figure 4E, *p* < 0.05). To test whether SNHG12 and NF-κB1 promoted IL-6R transcription, we measured IL-6R-promoter-dependent firefly luciferase reporter activity in cells overexpressing SNHG12 or NF-κB1. Overexpression of SNHG12 and NF-κB1 increased IL-6R-promoter-dependent luciferase activity and silencing NF-κB1 abolished luciferase activity induction caused by overexpressing SNHG12, which indicated SNHG12 promoted IL-6R transcription depended on NF-κB1 (Figure 4F, *p* < 0.05). Furthermore, we performed a ChIP assay which revealed that NF-κB1 directly bound the IL-6R promoter region, and overexpression of SNHG12 significantly increased NF-κB1 binding on IL-6R promoter (Figure 4G, *p* < 0.05). Consequently, IL-6R expression was dramatically downregulated by depletion of SNHG12 while overexpression of SNHG12 led to a marked increase in IL-6R expression (Figure 4H, *p* < 0.05). Collectively, our data revealed SNHG12 promoted IL-6R transcription by recruiting NF-κB1 to its promoter region through direct interaction with NF-κB1.

### SNHG12 inhibited T cell proliferation by facilitating the expression of PD-L1 in both ovarian cancer cells and M2 macrophages

**Figure 5:**
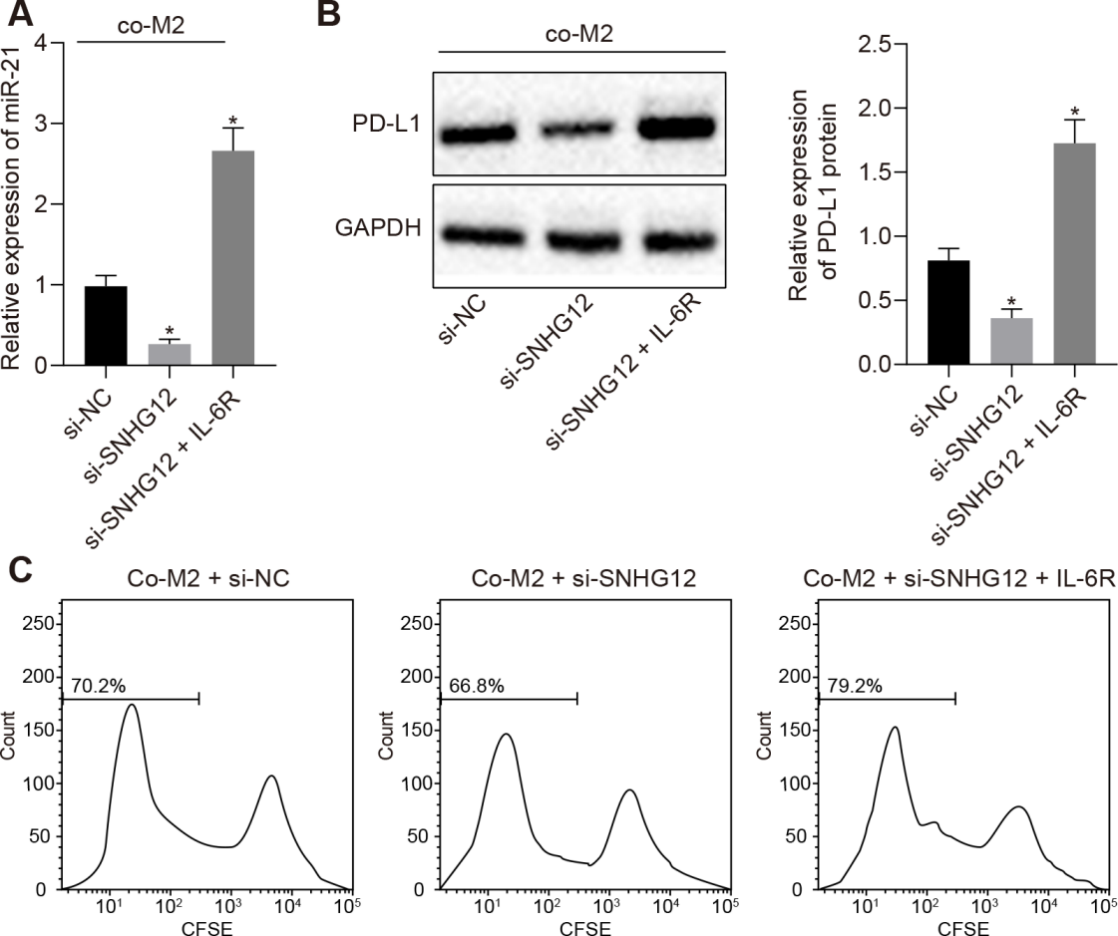
**SNHG12 suppresses T cell proliferation by upregulation of PD-L1.** (**A**) The expression of miR-21 determined by RT- qPCR. (**B**) protein levels of PD-L1 after SNHG12 depleted SKOV3 co-cultured with M2 macrophage determined by Western blotting analysis. (**C**) effect of SNHG12 depletion or IL-6R overexpression in SKOV3 co-cultured with M2 macrophages, on T cells proliferation determined by flow cytometry. * *p* < 0.05 *vs.* si-NC. Statistical comparisons were performed using unpaired t test when only two groups were compared or by Tukey’s test-corrected one-way ANOVA with when more than two groups were compared.

Based on the findings presented above, we next set out to determine whether SNHG12 played a role in ovarian cancer cell immune escape, mediated by the IL-6/miR-21 feedback loop. After co-culturing M2 macrophage and SKOV3 where SNHG12 has been silenced, we found that EVs from these SKOV3 contained lower amounts of miR-21. In addition, restoring IL-6R in SNHG12 depleted SKOV3 cells returned miR-21 expression to baseline (Figure 5A, *p* < 0.05). Consistent with the changes in miR-21 expression, the decrease in PD-L1 levels caused by depletion of SNHG12 could also be rescued by IL-6R overexpression (Figure 5B, *p* < 0.05). Furthermore, silencing SNHG12 abolished T cells growth inhibition caused by being co-cultured with SKOV3, which had been co-cultured with M2 macrophage in advance, and this process was also depended on IL-6R (Figure 5C, *p* < 0.05). These data demonstrated that SNHG12 was able to promote ovarian cancer immune escape by directly upregulating IL-6R and ultimately increasing PD-L1 levels.

### Silencing SNHG12 suppressed ovarian cancer immune escape in vivo

**Figure 6:**
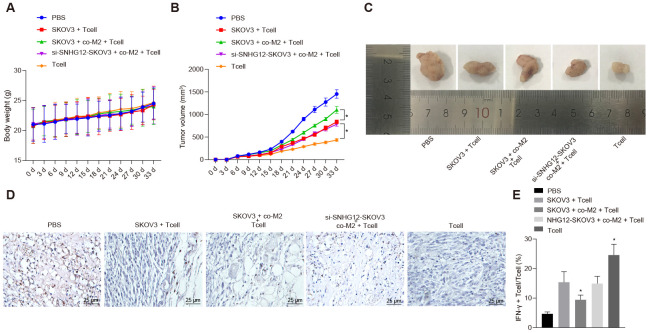
**SNHG12 depletion suppresses immune escape *in vivo.*** (**A**) Weight of mice recorded every 3 d. (**B**) Growth curve of transplanted tumor recorded every 5 d. (**C**) image of transplanted tumors (Scale = 1 mm). (**D**) CD3^+^ cells in transplanted tumors determined by IHC. (**E**) the rate of IFN-γ positive cells in transplanted tumors determined by flow cytometry. * *p* < 0.05 *vs.* SKOV3 + T cells. Statistical comparisons were performed using unpaired t test when only two groups were compared or by Tukey’s test-corrected one-way ANOVA with when more than two groups were compared. Variables were analyzed at different time points using Bonferroni-corrected repeated measures ANOVA.

**Figure 7:**
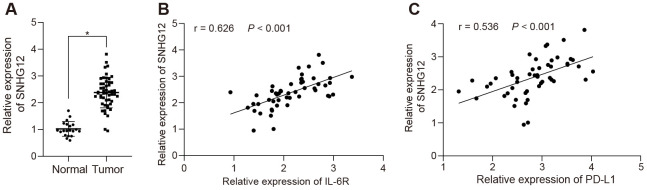
**SNHG12 is highly expressed in clinical ovarian cancer patients and positively correlates with IL-6R.** (**A**) the expression of SNHG12 in clinical ovarian cancer samples determined by RT-qPCR. (**B**) correlation between SNHG12 and IL-6 according to its ranks in related IHC results. (**C**) correlation of IL-6 and PD-L1 analyzed by IHC. Statistical comparisons were performed using unpaired t test. * *p* < 0.05 *vs.* normal tissue.

Next, we studied immune escape mediated by SNHG12 *in vivo*. SKOV3 cells were injected into nude mice subcutaneously and then, the mice were divided into six groups followed administration of the indicated T cells. Next, we monitored the weight of mice and the size of the tumor every three days. Notably, without any changes in the weight of the mice (Figure 6A), the tumor was much smaller in conditions where T cells were injected as opposed to PBS. However, mice injected with T cells co-cultured with SKOV3 which had been co-cultured with M2 macrophage showed largely restricted treatment efficiency. Strikingly, SNHG12 knockdown in SKOV3 cells restored the effect of T cells treatment on controlling tumor size (Figure 6B, *p* < 0.05). Consistently, tumor size showed similar trends (Figure 6C). Furthermore, to determine the number of tumor infiltrating T cells, we measured CD3 expression levels in tumors by IHC. Consistent with previous data, the CD3 positive cells were dramatically reduced in the tumors of mice injected with T cells co-cultured with SKOV3 which had been co-cultured with M2 macrophages, and this phenotype was mediated by SNHG12 (Figure 6D). Consistent with the CD3^+^ tumor infiltrating lymphocytes data, the percentage of IFN-γ in tumors from mice injected with T cells co-cultured with SKOV3 which had been co-cultured with M2 macrophage was dramatically reduced, and this phenotype was reversed after SNHG12 knock down (Figure 7E, *p* < 0.05). Taken together, SNHG12 inhibited T cell infiltration into SKOV3 tumors by enhancing the crosstalk between SKOV3 and M2 macrophages.

### SNHG12 was overexpressed in clinical ovarian cancer patients and positively correlated with IL-6R expression

To further investigate the expression levels of SNHG12 in clinical ovarian cancer samples, we determined the levels of the SNHG12 which was significantly upregulated in ovarian cancer clinical samples compared to adjacent normal tissues (Figure 7A, *p* < 0.05), suggesting that SNHG12 might be the cause of ovarian cancer immune escape. Notably, both PD-L1 and IL-6R were upregulated and positively correlated with SNHG12 in clinical samples (Figure 7B, 7C, *p* < 0.05). Collectively, our data suggested that SNHG12 might mediate immune escape in ovarian patient through upregulating PD-L1 by IL-6R.

## DISCUSSION

Increasing evidence shows that SNHG12 promotes the proliferation and metastasis of cancer cells [16, 17]. However, its role in the tumor immune escape in ovarian cancer remains unclear. Our study revealed that

**Figure 8:**
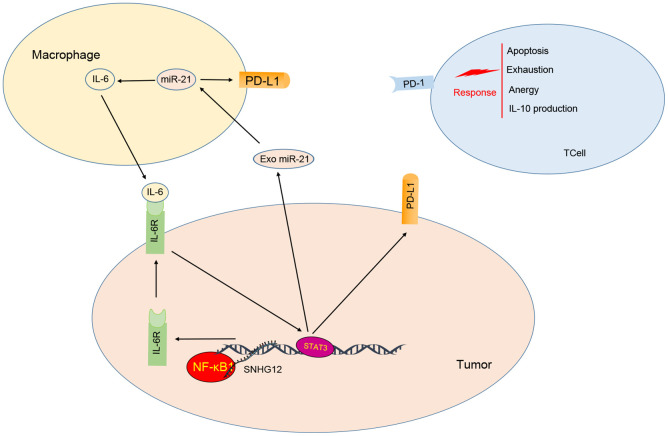
**Model for ovarian cancer immune escape mechanism: SNHG12 promotes IL-6/miR-21 crosstalk between ovarian cancer cells and M2 macrophages, through elevation of IL-6R *via* NF-κB1.**

IL-6 secreted from M2 macrophages stimulated the expression of PD-L1 and miR-21 in ovarian cancer cells. On the other hand, miR-21 containing EVs secreted by ovarian cancer cells are absorbed by M2 macrophage to further induce expression of IL-6 in a positive feedback loop. As a consequence, this crosstalk promotes escape from the immune system by ovarian cancer cells. In addition, the lncRNA SNHG12 enhanced this crosstalk by recruiting NF-κB1 to the IL-6R promoter increasing its expression and thus

amplifying IL-6 signaling. Through this mechanism, SNHG12 promoted PD-L1 expression on cancer cells, which in turn, suppressed the proliferation of T cells (Figure 8).

The tumor microenvironment is a dynamic and complex environment that is tightly controlled by tumor cells in response to extracellular signals [18] and miRNAs have been reported to mediate these processes [19]. Previous studies have shown that IL-6 from immune system cells can promote the expression of miR-21 and miR-29b in tumor cells which promotes their invasiveness ability; thus, as tumor cells stimulate IL-6 expression in immune cells they can promote their own tumor progression [11]. Meanwhile, other group reveals that glioma cells can reprogram microglia in part by transferring miR-21 through EVs that are secreted by tumor cells, thus changing the transcriptome and subsequent proliferative ability of microglia [20]. Our study also stated that M2 macrophages increased the expression of PD-L1 and miR-21 in ovarian cancer cells via induction of IL-6. Moreover, the upregulated miR-21 was packed into EVs, secreted by ovarian cancer which stimulated the secretion of IL-6 in M2 macrophage.

Many studies have shown that the IL-6 cytokine promotes tumor progression [21], as IL-6 is essential for inducting immune escape by upregulation of PD-L1 in a transcriptional or post-transcriptional manner [22, 23]. Previous data shows IL-6 can stabilize the PD-L1 protein by phosphorylation of JAK1 [23]. Meanwhile, another group reports that IL-6 also can facilitate the transcription of PD-L1 by STAT3 [22]. IL-6 recognition and activation of the downstream signaling pathway is dependent on the expression of its receptor, IL-6R. Interestingly, we analyzed clinical biopsy specimens and found that IL-6R and PD-L1 are correlated and highly expressed in ovarian cancer cells. Furthermore, IL-6R silencing significantly downregulated PD-L1 even in the presence of IL-6 indicating that IL-6R is essential for the IL-6/miR-21 crosstalk in order to promote PD-L1 expression.

Recent insights into the mechanism of action of lncRNAs have shown that these RNAs regulate gene transcription by changing mRNA stability, recruiting transcription factors to the promoters or, competitively binding to microRNAs [24–26]. Previous data show that SNHG12 can serve as a biomarker for poor diagnosis [27]. Collectively, the majority of studies focusing on SNHG12 suggest that it works by sponging off miRNAs. To the best of our knowledge, SNHG12 has not yet been reported as able to recruit transcription factors to a gene promoter. Here, we showed that SNHG12 facilitated IL-6R transcription by recruiting NF-κB1 to its promoter with an end result of expanding the miR-21/IL6 crosstalk and elevating PD-L1 expression. Furthermore, our study revealed SNHG12 was essential for the intercellular communication both *in vivo* and in *vitro*.

Taken together, our study shows that M2 macrophages via IL-6 secretion, elevated PD-L1 and miR-21 expression which then promoted immune escape in ovarian cancer cells. As a feedback signal miR-21 packed EVs facilitated the secretion of more IL-6 from M2 macrophage which strengthened the malignancy phenotype. IL-6R expression, being key mediator in transducing IL-6 signaling and inducing PD-L1 expression, was positively regulated by SNHG12. Notably, we identified the mechanism by which SNHG12 elevated IL-6R expression, namely through recruitment of NF-κB1 to the IL-6R promoter. Consistent with our *in vitro* data, SNHG12 was also found to be highly expressed in ovarian cancer clinical samples, suggesting that SNHG12 may be a promising biomarker for ovarian cancer diagnosis. Interestingly, IL6 blockade therapy has been shown to be less effective than dual cytokine blockade both in vitro and in vivo. This raises the question as to whether other cytokines from immune cells could elevate the expression of miR-21 and as feedback, EVs-derived miR-21 re-induced expression of IL-6 in immune cells. Thus, further investigation is required to explore the possibility of combining anti-IL-6 and EVs from ovarian cancer cells in a therapeutic approach.

## MATERIALS AND METHODS

### Study subjects

Human tumor biopsy specimens were collected from 51 patients who were diagnosed with ovarian cancer at Shanghai Ninth People’s Hospital, School of Medicine, Shanghai Jiaotong University from May 2017 to October 2018. Normal oviduct specimens were obtained from 20 patients with benign lesions. Part of these samples were used for total RNA extraction. Histological diagnosis and classification were done in accordance to the *World Health Organization Classification of Tumors of the Digestive System*.

### Immunohistochemistry

Formalin-fixed paraffin-embedded 5-μm tissue sections were deparaffinized in xylenes and rehydrated through an alcohol gradient. After antigen retrieval was performed, all sections were blocked at room temperature in avidin/biotin blocking buffer (Vector Laboratories) and then 3% BSA for 30 min. Next, the sections were incubated with primary rabbit antibodies against CD3 (ab135372, Abcam Inc., Cambridge, UK), IL-6R (ab228415; Abcam Inc.) and PD-L1 (ab9324, Abcam Inc.,) at room temperature for 60 min. Sections were rinsed twice in PBS, and protein staining was performed using a diaminobenzidine (DAB) substrate kit (Maixin Biotech, KIT-9710). Samples were counterstained with hematoxylin. Immunohistochemistry images were obtained using an upright microscope (Olympus BX51). Brown staining indicated the immunoreactivity of samples.

The positive expression of IL-6R and PD-L1 was confirmed when tumor cells showed brownish-yellow granules. The scoring for the percentage of positive cells was listed as follows: Positive cells ≤ 10% as negative; scored 0 regardless of staining intensity. Positive cells accounted for 11%-51% as positive expression; scored 2. Positive cells accounted for 51%-81% scored 3. Positive cells ≥ 81% scored 4. The grading for staining intensity: 1 point for weak intensity; 2 points for moderate intensity; 3 points for strong intensity. Cells were divided into 2 groups based on the total points.

### Cell culture

HEK-293T cells were purchased from the National Infrastructure of Cell Line Resource. SKOV3 and THP-1 were purchased from ATCC. HEK-293T and SKOV3 were cultured in Dulbecco’s Modified Eagle Medium (DMEM) (Gibco, Carlsbad, CA, USA) supplemented with 10% fetal bovine serum (FBS). THP-1 was cultured in Roswell Park Memorial Institute (RPMI)-1640 medium (Gibco, Carlsbad, CA, USA) supplemented with 10% FBS. All cells were maintained at 37°C in a saturated humidity atmosphere containing 95% air and 5% CO_2_. The cells were cultured to the sixth generation for later use.

### Macrophage M2 polarization and co-culture

THP-1 monocytes were differentiated into M2 macrophages by 12 h incubation with 100 ng/mL phorbol 12-myristate 13-acetate (PMA, Sigma-Aldrich Chemical Company, St Louis MO, USA, P8139) followed by incubation with 20 ng/ml of interleukin 4 (IL-4) and 20 ng/ml of interleukin 13 (IL-13). M2 macrophage marker CD206 (#91992; rabbit; Cell Signaling Technology, Danvers, USA) and CD11b (#49420; rabbit; Cell Signaling Technology) were detected by flow cytometry to make sure M2 macrophage have been successfully polarized.

In co-culture experiments, 1 × 10^5^ ovarian cancer cells were seeded in 6 Transwell chambers (#3450; Corning, NY, USA), and then put the inserts into 6-well plates containing differentiated THP-1 cells. The chamber was placed in a differentiated 6-well plate containing THP-1 cells and co-cultured for 48 h.

### Isolation and identification of T cells

Briefly, fresh peripheral blood mixed with heparin was centrifuged at 400 g for 5 min. PBS was used to dilute the rest blood cells and lymphocytes were isolated by using lymphocytes isolation buffer. Then, lymphocytes were cultured in 6-well plate containing anti-CD3/CD28 d-dimer antibody (STEMCELL Technologies) and maintained at 37°C in a saturated humidity atmosphere containing 95% air and 5% CO_2_. PE-CD3^+^ cells (130-113-129; 1:50; Miltenyi Biotec, Bergisch Gladbach, Germany) were isolated by flow cytometry [28].

### Detection of cell surface antigen

Cells were resuspended in staining buffer (BD biosciences). After being fixed and penetrated, cells were stained with PE-CD3 (130-117-139, 1:50, Miltenyi Biotec) and Pacific blue-IFNγ (BioLegend, #505817, Rat, 1:50). Samples were analyzed by flow cytometry (BD Immunocytometry System) and FlowJo software [29].

### Construction of stable knock down or overexpressed cell lines

The short hairpin RNA (shRNA) against SNHG12, IL-6, IL-6R, and miR-21, overexpression pbabe-SNHG12 and-NF-κB1 plasmids were purchased from Cyagen company (Suzhou, China). Packaging and indicated knock down or overexpression plasmids were co-delivered into HEK-293T cells and the medium was renewed after being transfected for 12 h. Virus was harvested after 48 h and filtered by 0.22 μm filter. After cells being infected with indicated virus, stably transfected cells were selected by 2 μg/mL puromycin (HY-B1743A, MCE, USA).

### Detection of T cell proliferation

CD3^+^ T cells were stimulated with 20 IU/mL IL-2, marked by carboxy fluorescein succinimidyl ester (CFSE) (S1076; Solarbio, Beijing, China) and co-cultured for 5 days with ovarian cancer cells in 96-well plate coated by anti-CD3/CD28 d-dimer antibody. Levels of CFSE were determined by flow cytometry [28].

### Enzyme-linked immunoassay (ELISA)

The medium supernatant was collected into tubes and stored at -20°C. The concentration of IL-6 was assessed by ELISA kit (ab178013, Abcam Inc.) following the instructions provided by the manufacturer.

### Isolation of EVs from supernatants and biological fluids

Briefly, cells were grown in DMEM medium with EVs-depleted 20% FBS. After 48 h, the media was collected and pre-cleared by centrifugation at 4500 g for 15 min to remove cell debris. EVs in supernatant were extracted using the Exoquick-TC manufacturer (EXOTC10A-1, Shanran biology company, Shanghai, China) and then observed under TEM (JEM-1010, JEOL, Tokyo, Japan).

Pelleted EVs used for Western blotting analyses were resuspended in RIPA lysis buffer supplemented with protease inhibitors cocktail. Protein concentration of isolated EVs was determined by the BCA assay according to the manufacturer’s instruction (Pierce BCA Protein Assay Kit, ThermoFisher Scientific). The markers of EVs, TSG101 (ab125011, 1:1000, Abcam Inc.), CD63 (ab134045, 1:1000, Abcam Inc.), and histone (ab1791, 1:1000, Abcam Inc.), were analyzed by western blotting [30].

### Immunofluorescence

EVs from SKOV3 were stained by CFSE at 37°C for 15 min and then washed by PBS for once. Following, M2 macrophage were co-cultured with CFSE-marked EVs and observed using a Confocal laser microscope (Carl Zeiss AG, Germany) every 12 h, 24 h, and 48 h [31].

### Reverse transcription quantitative polymerase chain reaction (RT-qPCR)

Total RNA was extracted using TRIzol (Invitrogen). Complementary DNA (cDNA) for miRNA detection was obtained as Tailing Reaction (b532451, Sangon, Shanghai, China) following the instructions provided by the PolyA tail test kit (B532451; Sangon, Shanghai, China). other cDNA was synthesized using Reverse Transcription cDNA synthesis kit (K1622, Reanta, Beijing, China) and then RT-qPCR was performed by GoTaq qPCR master mix (Promega). Primers were listed in Table 1. The miR-21 level was normalized to U6 and other mRNA levels to GAPDH. Results were calculated by using 2^-ΔΔCT^ method [32].

**Table 1:** Primer sequences used for RT-qPCR.

**Targets**	**Primer sequence**
miR-21	F: 5’- ACACTCCAGCTGGGTAGCTTATCAGACTGA-3’
	R: 5’- TGGTGTCGTGGAGTCG-3’
U6	F: 5’- GCTTCGGCAGCACATATACTAAAAT-3’
	R: 5’- CAGTGCGTGTCGTGGAGT-3’
Lnc-SNHG12	F: 5’- GGTGCTCCAGGCAATAACT-3’
	R: 5’- CTCCCATACAGTCCGAACAT-3’
GAPDH	F: 5’- TCACCAGGGCTGCTTTTAAC-3’
	R: 5’- GACAAGCTTCCCGTTCTCAG-3’

Notes: MiR-21, MicroRNA-21; Lnc-SNHG12, Long-non coding RNA-SNHG12; ZNF217; GAPDH, Glyceraldehyde-3-phosphate dehydrogenase.

### Fluorescence in situ hybridization (FISH)

The subcellular localization of SNHG12 was determined by Ribo^TM^ lncRNA FISH Probe Mix (Red) (Ribobio, China). Briefly, cells were seeded in 6-well plates where a cover glass was placed in advance. After reached 80% confluence, cells were fixed and stained according to the instructions provided by the manufacturer. A fluorescent microscope was used to observe the subcellular localization of SNHG12.

### Dual luciferase reporter assay

IL-6R promoter region was cloned into pmirGLO luciferase vector (Promega, WI, USA). And then, IL-6R was introduced into HEK-293T in the presence of indicated combination of sh-NF-κB1, NF-κB1, sh-SNHG12, or SNHG12. And then luciferase activity was measured by a luciferase assay reagent (Promega, Fitchburg, WI, USA), relative to that of renilla luciferase.

### RNA immunoprecipitation (RIP) assay

RIP was performed using the EZ-Magna RIP kit (Millipore) according to the manufacturer’s instructions. Briefly, we lysed SKOVE3 cells by Radio-Immunoprecipitation Assay (RIPA) containing mixture of protease and phosphatase inhibitor (Sigma-Aldrich Chemical Company, St Louis MO, USA). The magnetic beads (Invitrogen) were pre-incubated with antibody anti-argonaute RISC catalytic component 2 (AGO2) (ab32381, 1 : 30, Abcam Inc.) or anti-rabbit immunoglobulin G (IgG) (ab6721, 1 : 30, Abcam Inc.) for 30 min. Next, the lysate was immunoprecipitated with magnetic beads at 4°C overnight. RNA was purified from RNA-protein complex, and analyzed by RT-qPCR.

### Chromatin immunoprecipitation (CHIP) assay

Cells were treated with formaldehyde to create protein-DNA crosslinks, and the crosslinked chromatin was extracted and sheared by sonication. Protein A/G beads were precleared and blocked with 1% salmon sperm DNA and 1% BSA. Total sheared chromatin was used for immunoprecipitation with either normal rabbit IgG (ab109489, 1:300, Abcam Inc.) or NF-κB1 (#13586, Cell Signaling Technology, USA) antibody. The immunoprecipitants were washed 5 times, pelleted by centrifugation, and then heated at 65°C for 4 h for decrosslinking. The products were then analyzed by RT-qPCR.

### Protein extraction and quantification

Protein extraction was performed using the protease inhibitor-contained RIPA buffer (R0010, Solarbio, Beijing, China). The protein sample was separated using freshly-prepared SDS-PAGE, electrotransferred onto PVDF membranes, and probed with primary antibodies: PD-L1 (ab228415, 1: 1000), IL-6R (ab128008, 1:1000), and β-actin (ab8226, 1:20000) (Abcam Inc.). Immunoblotting was visualized with goat anti-rabbit IgG (Transgene biology CO., Beijing, China), enhanced chemiluminescence detection reagents and captured under the Bio-Rad image system (Bio-Rad, USA). Gray value of the target protein bands was quantified using Quanity One software, with β-actin used for normalization.

### Tumor xenograft

Twenty-five five-weeks-old NOD/SCID female nude mice were obtained from SLAC CO. (Shanghai, China) and maintained in a specific pathogen-free environment. SKOV3 cells (2 × 10^6^ cells/mice) were transfected with si-NC or si-lncRNA-SNHG12 were subcutaneously injected into the flanks of mice.

Ten days post injection of tumor cells, PBS or T cells (5 × 10^6^) from indicated conditions was injected intraperitoneally into mice. The vertical diameter of the tumor was recorded every 5 days. The formula for calculating tumor volume is as follows: tumor volume (mm^3^) = a × b^2^ × 0.5 (a represents the largest diameter, b, shortest diameter, and 0.5, a constant for calculating the ellipsoid volume).

### Statistical analysis

All data were processed using SPSS 22.0 statistical software (IBM Corp. Armonk, NY, USA). Data are shown as the mean ± standard deviation from at least three independent experiments. Unless otherwise noted, statistical comparisons were performed using unpaired t test when only two groups were compared or by Tukey’s test-corrected one-way analysis of variance (ANOVA) with when more than two groups were compared. Variables were analyzed at different time points using Bonferroni-corrected repeated measures ANOVA. *p* < 0.05 was considered as a level of statistical significance.

### Ethics statement

All experimental procedures involving animals were approved by the Institutional Animal Care and Use Committee of Shanghai Ninth People’s Hospital, School of Medicine, Shanghai Jiaotong University (Approval No. 201910053) on 2^nd^ November, 2019. The study was approved by the ethics committee of Shanghai Ninth People’s Hospital, School of Medicine, Shanghai Jiaotong University (Approval No. 201705139) on 20^th^ April, 2017, and all 51 patients signed informed consents.
